# Elevated Serum Gas6 Is a Novel Prognostic Biomarker in Patients with Oral Squamous Cell Carcinoma

**DOI:** 10.1371/journal.pone.0133940

**Published:** 2015-07-24

**Authors:** Tao Jiang, Guoxia Liu, Lin Wang, Hongchen Liu

**Affiliations:** 1 Department of Stomatology, Chinese PLA General Hospital, Beijing, People’s Republic of China; 2 Department of General Dentistry, Jinan Stomatological Hospital, Jinan, Shandong, People’s Republic of China; 3 Department of Clinical Laboratory, Jinan Stomatological Hospital, Jinan, Shandong, People’s Republic of China; Duke Cancer Institute, UNITED STATES

## Abstract

**Objective:**

This study explored the level and clinical significance of serum Gas6 in patients with oral squamous cell carcinoma (OSCC).

**Methods:**

A total of 128 OSCC patients and 145 normal controls were selected. Enzyme-linked immunosorbent assay was used to detect Gas6 concentration in sera from the OSCC patients and controls. The correlations of serum Gas6 concentration and clinicopathological characteristics of OSCC patients were assessed, and the prognostic significance of serum Gas6 was evaluated with a Kaplan–Meier curve and log-rank test.

**Results:**

The results showed that serum Gas6 concentration was significantly higher in OSCC patients than in controls (P < 0.05). OSCC patients with late TNM stage (III, IV) had a relatively high serum Gas6 concentration compared with those with early stage (I, II) (P < 0.01) and patients with poorly differentiated tumors had a higher level of serum Gas6 than those with well-differentiated tumors (P < 0.01). Multivariate logistic regression analysis demonstrated that high serum Gas6 was an independent risk factor for lymph nodal metastases in OSCC patients (OR = 2.79, 95% CI: 1.72–4.48). For predicting OSCC development, ROC curve analysis showed a sensitivity of 0.63 with a specificity of 0.92 (AUC = 0.79, 95% CI: 0.74–0.85). Cox analysis revealed that high serum Gas6 was an independent biomarker for predicting poor overall survival in OSCC patients (HR = 2.07, 95% CI: 1.79–3.62). In addition, we found that Gas6 expression was increased in OSCC tissues and it may significantly decrease E-cadherin expression, and increase P-cadherin and N-cadherin expression, in OSCC cells. Further, Gas6 could promote the migratory and invasive ability of OSCC cells *in vitro*.

**Conclusion:**

Taken together, these results suggest that Gas6 increases the metastatic capacity of OSCC cells and serum Gas6 could be a candidate biomarker for diagnostic and prognostic use in OSCC patients.

## Introduction

Oral squamous cell carcinoma (OSCC) is one of the most lethal malignancies worldwide and is also the most common oral cancer [1]. OSCC is accompanied by frequent metastasis, high recurrence and poor prognosis [2]. It has been estimated that worldwide, more than 100,000 deaths result from OSCC annually, making it a significant burden on society and public medical systems [1]. As a multifactorial disease, OSCC is primarily associated with several environmental factors such as tobacco and alcohol use, chronic inflammation, and viral infections (human papillomavirus). Genetic susceptibility has also been implicated in OSCC development [3–5]. Until now, OSCC diagnostic procedures and therapeutic decisions have been based mainly on standard histological evaluation. This method is invasive and insufficiently sensitive for early diagnostic purposes. Therefore, a noninvasive method for diagnostic and prognostic use in OSCC could be an important clinical advancement in the management of OSCC patients.

The TAM (Tyro3/Axl/Mer) receptors are a family of receptor tyrosine kinases, characterized by adhesion molecule-like extracellular domains and a conserved sequence within the kinase domain [6]. TAM receptors play an important role in hemostasis and inflammation, and a previous study has reported that TAM receptor signaling could influence cell proliferation, survival, adhesion and migration [7]. Elevated Axl activity and overexpression were observed in several types of human cancer, and were related to invasiveness and metastasis [8, 9]. Mer was reported to be overexpressed or ectopically expressed in a variety of cancers and potentially leading to the activation of several canonical oncogenic signaling pathways, including mitogen-activated protein kinase and phosphoinositide 3-kinase pathways [10]. Additionally, Tyro3 knockdown may induce the proliferation suppression and cell cycle arrest in cancer cells [11]. All these findings have demonstrated TAM receptor signaling, physiologic and oncogenic functions.

As the ligands for TAM receptors, the vitamin K-dependent proteins Gas6 and Protein S may activate TAM signaling and enhance many essential biological functions for cancer formation and progression through TAM signaling pathways, including cell transformation, proliferation, angiogenesis, invasion and migration [12, 13]. Researchers have found that Gas6 expression is increased in several different cancers, potentially a biomarker for cancer patients [14, 15]. However, the expression level of Gas6 in OSCC remains unclear. Our study detected the expression level of Gas6 in OSCC patients using enzyme-linked immunosorbent assay (ELISA), real-time polymerase chain reaction (RT-PCR) and western blotting, and further analyzed the potential diagnostic and prognostic ability of serum Gas6, in OSCC patients.

## Materials and Methods

### Study subjects

A total of 128 consecutive OSCC patients and 145 healthy controls were recruited. The 128 OSCC patients were from the department of Stomatology, Chinese PLA General Hospital (Beijing, China), diagnosed with OSCC between June 2009 and September 2011. OSCC was confirmed in all cases by histological examination of tissue from biopsy or resected specimens. A total of 145 healthy controls, unrelated to the OSCC patients, were matched by gender and age to individual OSCC patients during the same period. The study was approved by the Ethics Committee of the Chinese PLA General Hospital and written informed consent was obtained from all participants.

### Sera samples and data collection

From all OSCC patients, 2 ml venous blood was collected before surgery. Blood samples were centrifuged at 3000 g for 10 min, and sera samples were carefully removed and stored in -80°C prior to use. All clinical and laboratory data reported were obtained at the time of serum sampling. Histological cancer grade and tumor stage of each patient were evaluated according to World Health Organization standards and the American Joint Committee on Cancer System. A total of 97 OSCC patients were recruited in the follow-up period after surgery and follow-up began from the date of surgery. The final follow-up was on November 20, 2014, with follow-up time ranging from 4 to 54 months. Sera samples from normal controls were also collected using the same method.

### Enzyme-linked immunosorbent assay

The level of serum Gas6 was measured using a commercial ELISA kit (GenWayBio, San Diego, USA) according to the manufacturer's protocol. In brief, 96-well plates were coated with human Gas6 monoclonal antibody. The sera samples from all participants and Gas6 protein standard were diluted with sample dilution buffer and added to the plates. The plates were then incubated at room temperature for approximately 2 h. Following this, a secondary biotin-conjugated antibody was utilized for measurement, together with a streptavidin-peroxidase/HRP-streptavidin conjugate and TMB solution. The plates were washed five times between each step, for 10 min each time. Finally, 2N sulphuric acid was used to stop the reaction. The absorbance at 450 nm was measured by a micro-plate reader system. A standard curve was drawn by serial dilution of recombinant Gas6 protein standard. According to the standard curve and the average optical density from triplicate samples, serum Gas6 concentration from each participant was obtained.

### RNA collection and quantitative real-time polymerase chain reaction

Total RNA was isolated from tumor and tumor-adjacent tissue using Trizol (Invitrogen, CA, USA) according to the manufacturer’s instructions. Approximately 1 μg of total RNA from each sample was used to synthesize cDNA using a reverse transcription kit (Takara, Japan). The reverse transcription reaction was carried out at 35°C for 10 min, followed by 95°C for 5 s. Quantitative real-time polymerase chain reaction (qRT-PCR) was performed using the ABI7500 system (ABI, Foster City, CA, USA). The final volume of the qRT-PCR system was 20 μl, including 10 μl of SYBR Green PCR Master Mix (ABI, Foster City, CA, USA), 1 μl of cDNA, 0.5 μl of forward primers, 0.5 μl of reverse primers and 8 μl ddH_2_O. qRT-PCR condition was set as 95°C for 15 s, 60°C for 30 s and 72°C for 30 s, for 40 cycles. Gene expression was measured relative to GAPDH as an internal control. The equation 2^−ΔCt^ (ΔCt = Ct_Gas6_−Ct_GAPDH_) was used to calculate the relative expression level of Gas6. The primers were: 5’-ATCAAGGTCAACAGGGATGC-3’ (forward primer), 5’-CTTCTCCGTTCAGCCAGTTC-3’ (reverse primer) for Gas6, 5’-CCATGTTCGTCATGGGTGTGAACCA-3’ (forward primer), and 5’-GCCAGTAGAGGCAGGGATGATGTTC-3’(reverse primer) for GAPDH, respectively.

### Protein isolation from tumor tissue and western blotting

Frozen tissue from tumor, tumor-adjacent and YD38 cells was homogenized with RIPA buffer using a glass homogenizer on ice. The homogenate was centrifuged at 12 000 g at 4°C for 30 min to remove cell debris, and the protein concentration was then determined by BCA assay kit (Bioyetime Biotech, China). The proteins were separated by 10% SDS-PAGE gels. Separated proteins were transferred to polyvinylidene difluoride membranes (Millipore, Billerica, MA, USA). The membrane was blocked with 5% skimmed milk in TBST, and then incubated with human Gas6 antibody (Mouse, RD system, USA) and GADPH antibody (Rabbit, Abcam, USA) at 4°C overnight. Subsequently, horseradish peroxidase-conjugated anti-mouse IgG and anti-rabbit IgG were used as secondary antibodies. The signals were captured by a CCD camera-based image system (Bio-Rad, CA, USA) using HRP Chemiluminescent kit (Yuanchuang lnc, China)

### Cell culture

Human OSCC cell line (YD38) was cultured in RPMI 1640 supplemented with 10% fetal bovine serum (Hyclone, Logan, UT, USA), 100 units/mL penicillin, and 100 μg/mL streptomycin (Gibco, USA). Cells were cultured in a humidified atmosphere of 5% CO_2_ at 37°C. Both Gas6 recombinant protein (hrGas6) and block/neutralizing Gas6 antibody (B/N anti-Gas6) were purchased from RD System Company. YD38 cells were incubated with hrGas6 and B/N anti-Gas6 according to the instructions of the manufacturer and the cells were harvested at three time points (6, 12 and 24 h), The proteins were also isolated using this method.

### Scratch wound healing assay and cell transwell invasion assay

To evaluate the effect of Gas6 on the metastatic capability of OSCC cells, scratch wound healing assay and cell transwell invasion assay were used to determine the migration and invasion of YD38 cells. We used a pipette tip to scrape cell layers when YD38 cells reached 90% confluence. Cells were then cultured in a humidified atmosphere of 5% CO_2_ at 37°C for 24 h and photographs were taken at 12 and 24 h. For invasion assay, YD38 cells were resuspended in serum-free RPMI 1640. YD38 cells were then seeded onto the upper portion of transwell matrigel invasion chambers (Costar, USA). The lower compartment contained RPMI 1640 with 10% FBS. Both non-invading cells and gel were discarded from the upper chamber after incubation in a humidified atmosphere of 5% CO_2_ at 37°C for 12 and 24 h. Finally, the invading YD38 cells on the filters were fixed with methanol and stained with crystal violet solution.

### Statistical analysis

An unpaired Student’s t-test was used to compare differences in clinical data, serum Gas6 concentration between OSCC patients and normal controls when continuous data were normally distributed. Otherwise, the Mann—Whitney U-test was used. A paired Student’s t-test was used to analyze Gas6 mRNA expression level between tumor tissue and tumor-adjacent tissue. Densitometric analysis of western blot was performed with ImageJ software. Survival probabilities were estimated with Kaplan—Meier analysis and significant differences were analyzed with the log-rank test. Multivariate analysis of prognostic factors was conducted using a Cox regression analysis model. All statistical analyses were performed in SPSS 18.0 (SPSS Inc., Chicago, IL). P < 0.05 was considered statistically significant.

## Results

### Serum Gas6 level was significantly increased in OSCC patients

**Fig 1:**
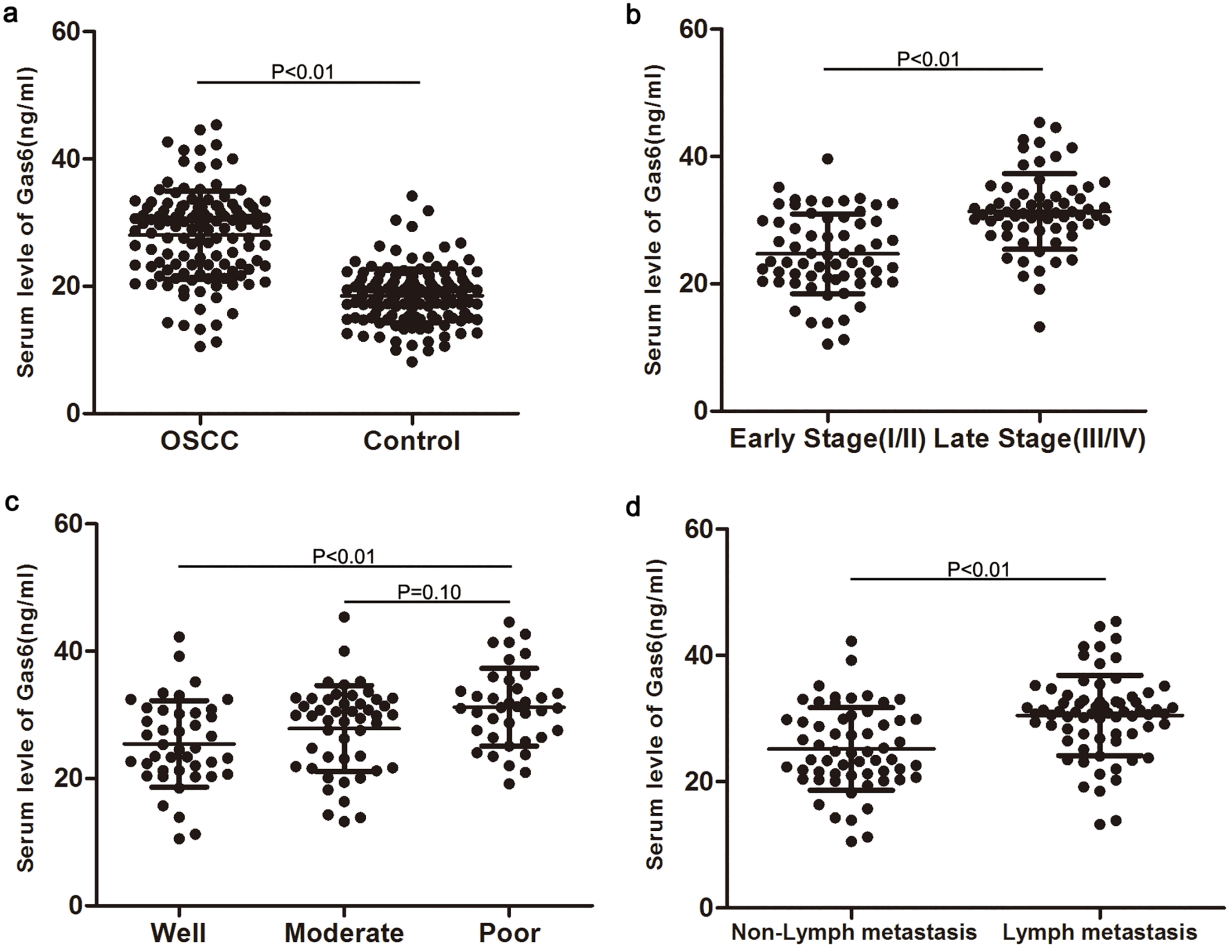
Serum Gas6 level for 128 OSCC patients and 145 normal controls (a); serum Gas6 level in OSCC patients with differing TNM stages (b); serum Gas6 level in OSCC patients with differing pathological differentiation (c); serum Gas6 level in OSCC patients with and without lymph nodal metastases (d).

The characteristics of OSCC patients and normal controls are described in S1 Table. Gas6 concentration in sera from OSCC patients and normal controls was shown in Fig 1a. The results showed that serum Gas6 level was significantly higher in OSCC patients than controls (28.05 ± 6.91 ng/ml vs. 18.50 ± 4.19 ng/ml, P < 0.01).

### Serum Gas6 level was associated with TNM stage and tumor differentiation

The correlations of Gas6 concentration and clinicopathological features in OSCC patients were analyzed further. OSCC patients with late TNM stage (III, IV) had higher serum Gas6 concentration than those with early stage (I, II) (P < 0.01; Fig 1b). OSCC patients with poorly differentiated tumors also had a relatively high level of Gas6 compared with those with well-differentiated tumors (P < 0.01), but not those with moderately differentiated tumors (P = 0.10) (Fig 1c). Smoking and alcohol use were clearly defined potential risk factors contributing to initiation and promotion of OSCC. Associations of serum Gas6 level with gender, smoking and alcohol use were evaluated but no difference was observed (P = 0.20, P = 0.32, P = 0.47, respectively).

### Serum Gas6 level could predict nodal metastases and TNM late stage

**Fig 2:**
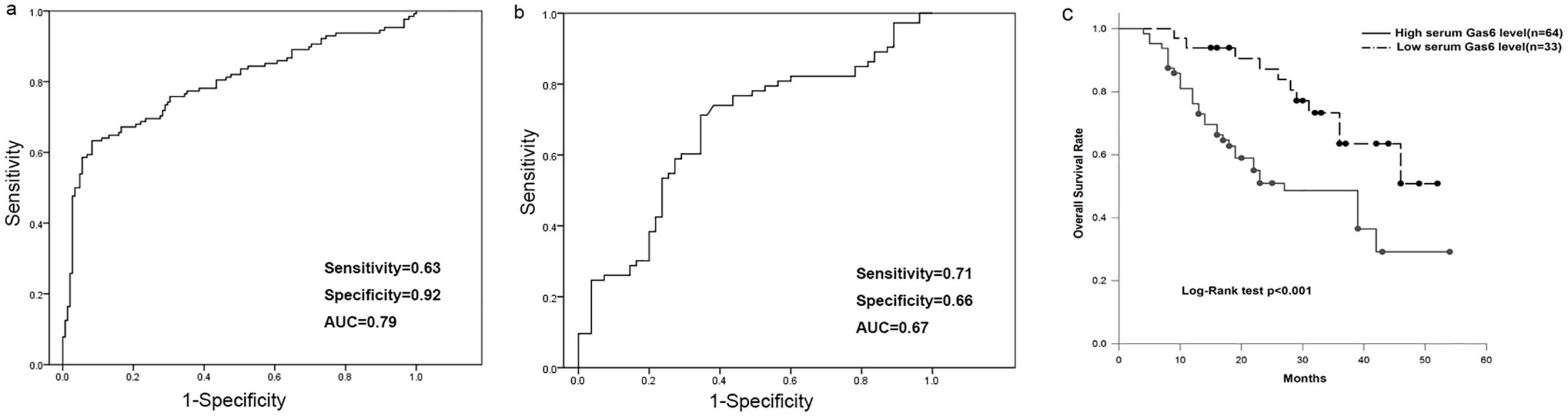
Receiver operating characteristic curves of serum Gas6 for differentiating OSCC patients from normal controls (a); receiver operating characteristic curves of serum Gas6 for differentiating OSCC patients with late stage (III, IV) from those with early stage (I, II) (b); Kaplan—Meier overall survival curve of OSCC patients in correlation with serum Gas6 level (c).

Serum Gas6 level was significantly higher in OSCC patients with lymph nodal metastases (P < 0.01; Fig 1d). Multivariate logistic regression analysis showed that high serum Gas6 was an independent risk factor for lymph nodal metastases in OSCC patients after adjusting for gender, age, smoking and alcohol use (OR = 2.79, 95% CI: 1.72–4.48). To further evaluate whether serum Gas6 level could serve as a diagnostic biomarker for OSCC patient an ROC curve was drawn by plotting the sensitivity versus the specificity for different Gas6 thresholds. For predicting tumor development, ROC analysis showed a sensitivity of 0.63 and specificity of 0.92 (AUC = 0.79, 95% CI: 0.74–0.85; Fig 2a) when a threshold value of 26.3 ng/ml was used. For differentiating patients at late stages (III, IV) from those at early stages (I, II), ROC analysis gave a sensitivity of 0.71 (specificity of 0.66) when a threshold value of 27.6 ng/ml was used (AUC = 0.67, 95% CI: 0.58–0.77; Fig 2b).

### Serum Gas6 level was an independent prognostic biomarker for overall survival in OSCC patients

To further assess whether serum Gas6 level could predict OSCC prognosis, we conducted a survival analysis. The disease-free survival time was measured as the time from the day of surgery until the date of reoccurrence/death or the last follow-up, to represent disease progression. The average level of serum Gas6 was used as the threshold and 97 OSCC patients were divided into two main groups: high serum Gas6 level group (>28.05 ng/ml; n = 64) and low serum Gas6 level group (<28.05 ng/ml; n = 33). Kaplan—Meier analysis revealed a survival difference between these two groups that was statistically significant (P < 0.001; Fig 2c), suggesting OSCC patients with a high serum Gas6 level had significantly poorer survival than those with a low serum Gas6 level. To determine whether serum Gas6 level was an independent risk factor for prognosis, Cox univariate and multivariate hazard regression models were then used, shown in Table 1. For univariate analysis, high serum Gas6, late TNM stage and poor differentiation were significantly associated with poor prognosis. In addition, multivariate Cox analysis revealed that high serum Gas6 level was an independent factor for poor overall survival in OSCC patients (HR = 2.07, 95% CI: 1.79–3.62).

**Table 1:** Univariate and Multivariate Cox Proportional Hazard Analysis of Prognostic Factors for Overall Survival Rates of OSCC Patients.

Factors	Categories	Univariate	Multivariate	
		P	HR(95%CI)	P
Age	>53/<53	0.33	0.76(0.44–1.31)	0.32
Gender	Male/Female	0.20	0.67(0.32–1.39)	0.28
Smoking	Smoker/Non-smoker	0.23	1.47(0.71–3.04)	0.29
Alcohol use	Drinker/Non-drinker	0.85	0.84(0.43–1.65)	0.31
Tumor differentiation	Well/Moderate +Poor	<0.05	0.66(0.30–1.44)	0.29
Lymph nodal metastases	Yes/No	<0.01	2.82(1.24–3.22)	<0.01
TNM stage	III+IV/I+II	<0.01	3.33(1.01–5.46)	<0.01
Serum Gas6 level	High/Low	<0.01	2.07(1.79–3.62)	<0.05

HR: Hazard ratio; 95% CI: 95% Confidence interval; TNM: Tumor nodal metastases.

### Gas6 expression was increased in OSCC tumor tissue

**Fig 3:**
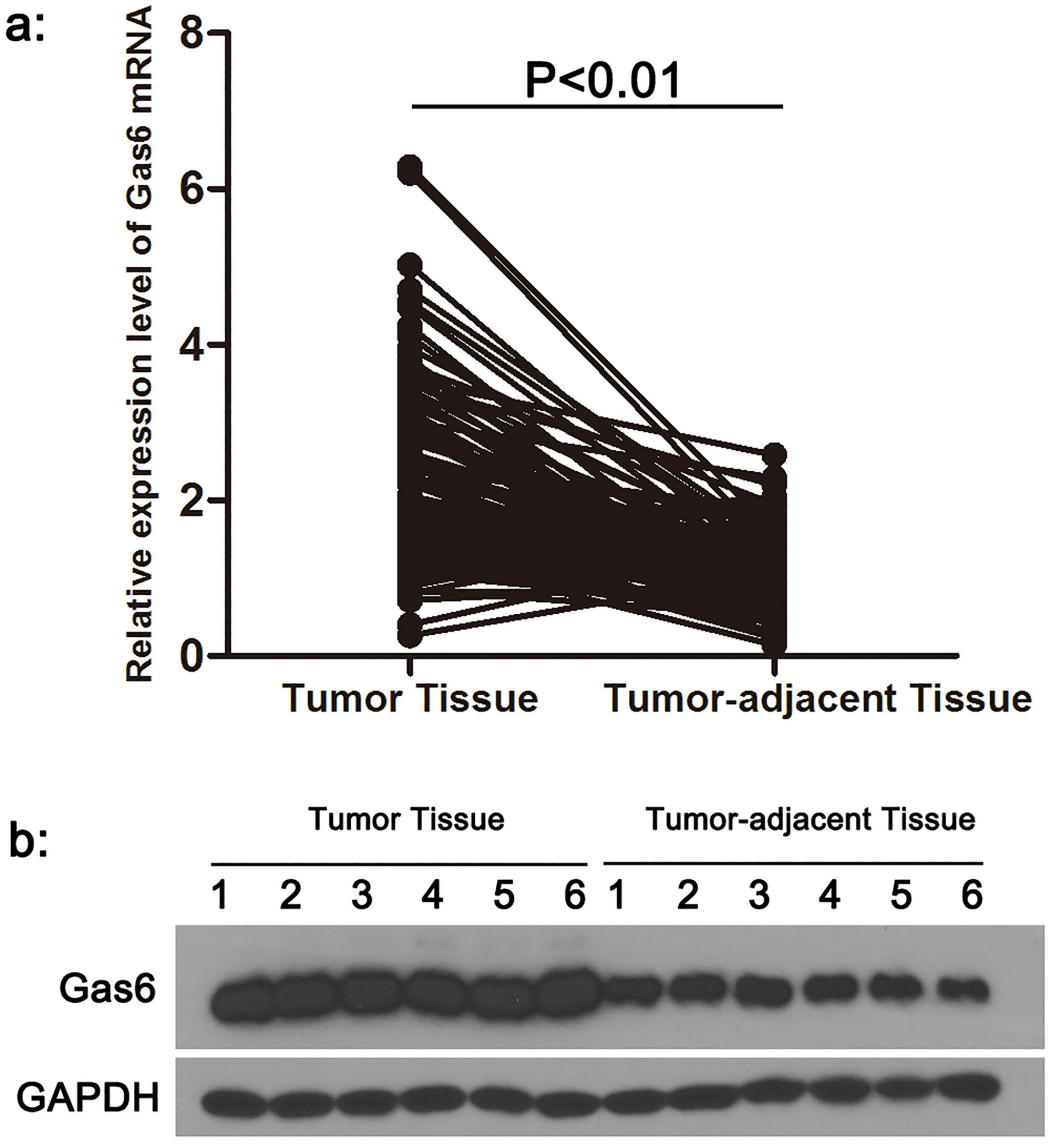
The mRNA and protein expression level of Gas6 between tumor tissue and tumor-adjacent tissue in OSCC patients (a, b).

To locate the source of high serum Gas6, we compared the mRNA expression level of Gas6 in tumor and tumor-adjacent tissue from OSCC patients. As shown in Fig 3a, the level of Gas6 mRNA in tumor tissue from OSCC patients was significantly increased when compared with tumor-adjacent tissue (P < 0.01). We then detected the protein level of Gas6 in OSCC tissue. Consistent with the mRNA level, comparison of Gas6 protein level between tumor tissue and tumor-adjacent tissue showed that the protein level of Gas6 in tumor tissue was also higher than in tumor-adjacent tissue (Fig 3b, S2 Fig).

### Gas6 could promote epithelial-mesenchymal transition in OSCC cell

**Fig 4:**
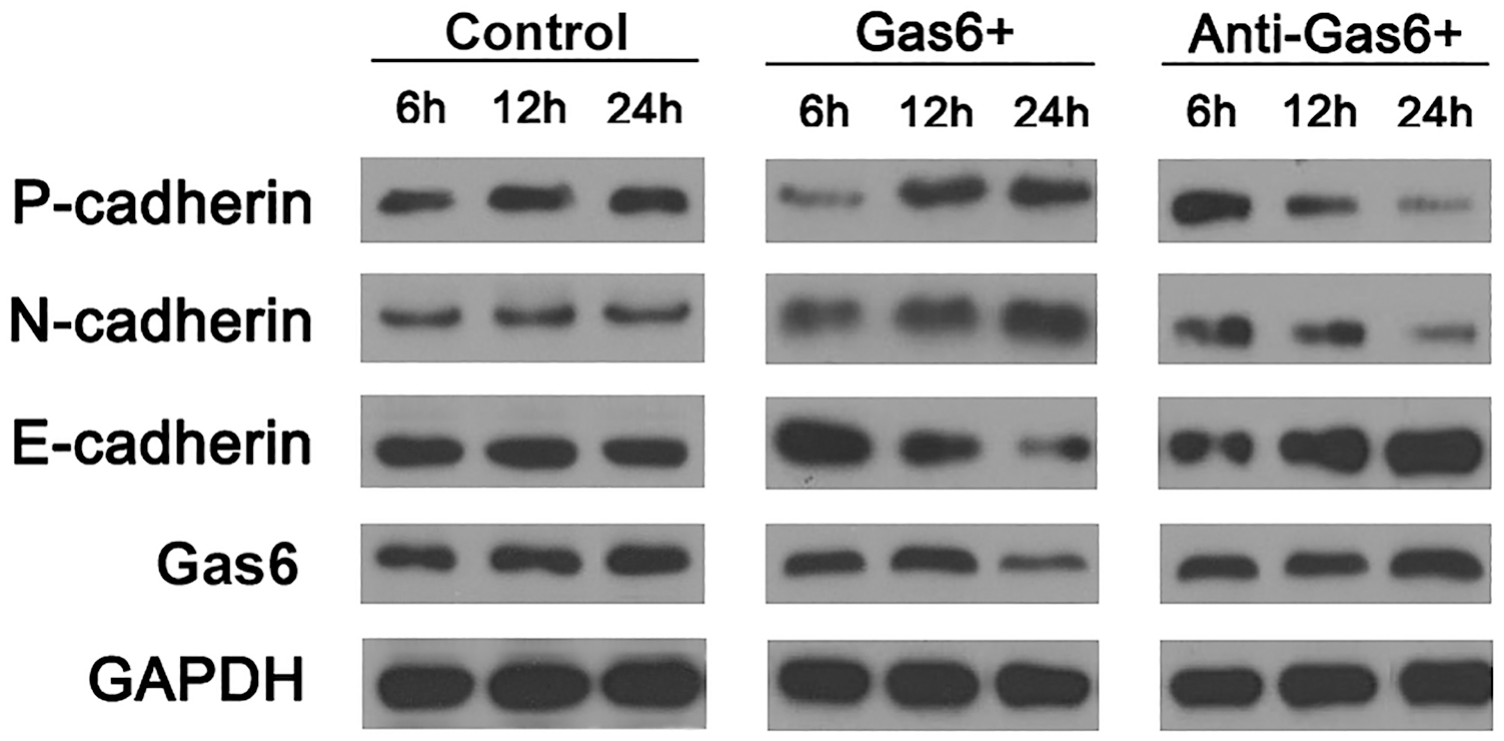
P-cadherin, N-cadherin, and E-cadherin expression in YD38 cells upon hrGas6 and block/neutralizing Gas6 antibody incubation.

The results demonstrated that high serum Gas6 level was significantly associated with tumor nodal metastases. To detect whether Gas6 could influence the expression level of epithelial-mesenchymal transition (EMT) genes in OSCC cell lines (YD38 cells), YD38 cells were incubated with human recombinant Gas6 protein (hrGas6) for 24 h. We then used a block/neutralizing Gas6 antibody to suppress Gas6 activation. The expression levels of epithelial marker E-cadherin, and mesenchymal markers P-cadherin and N-cadherin, were analyzed by western blotting. After incubating with hrGas6, the expression level of E-cadherin was significantly decreased, and P-cadherin and N-cadherin were significantly increased. Conversely, the epithelial marker was increased and mesenchymal markers were decreased after lowering Gas6 activation by anti-Gas6 antibody (Fig 4, S3 Fig).

### Gas6 could promote migration and invasion of OSCC cells in vitro

**Fig 5:**
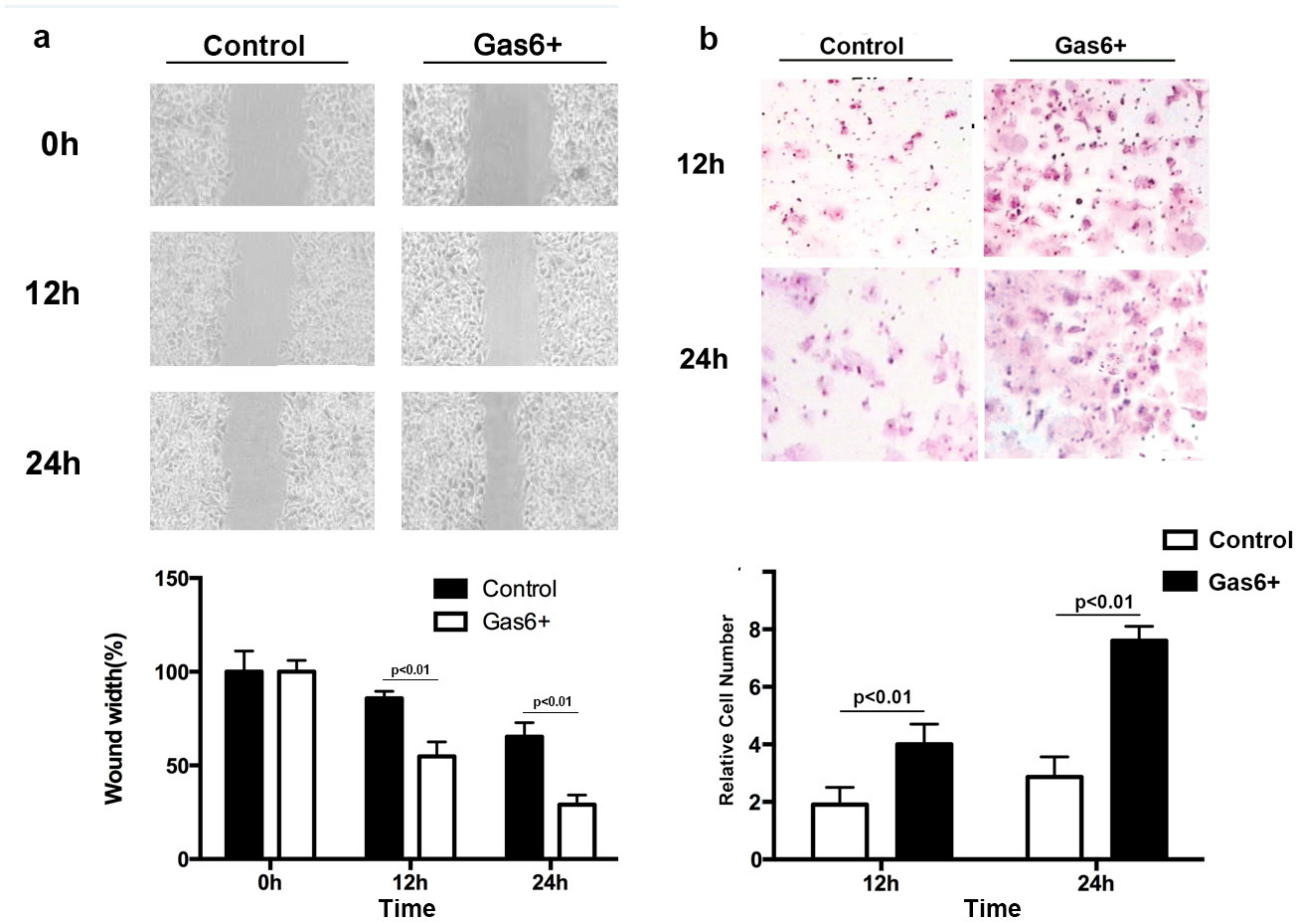
The influence of hrGas6 stimulation on metastatic capacity of YD38 cell. YD38 cell migration by scratch wound healing assay (a); YD38 cell invasion by cell transwell assay (b).

To further evaluate the cytological effect of Gas6 on movement ability of OSCC cells the scratch wound healing assay was used. As shown in Fig 5a, the YD38 cells migrated faster than controls upon hrGas6 incubation (P < 0.01). We next examined the influence of Gas6 on the invasion of OSCC cells and cell transwell invasion assay was used to determine the metastatic capacity of YD38 cells. As shown in Fig 5b, the YD38 cells had significantly increased invasive ability upon Gas6 incubation. The relative number of invasive YD38 cells was significantly higher compared with control (P < 0.01).

## Discussion

Our study demonstrated that serum Gas6 level was significantly increased in sera from OSCC patients and Gas6 expression was also increased in tumor tissues. Patients with late TNM stage and poorly differentiated tumors had relatively high levels of serum Gas6. Elevated Gas6 level was also predictive for nodal metastases and late cancer stages, and could reflect poor prognosis for OSCC patients. Additionally, this study confirmed that Gas6 was involved in EMT in OSCC cells and promoted cell migration and invasion.

Until now, OSCC diagnostic procedures and therapeutic decisions have been based mainly on histological evaluation, and this has not changed for decades. Serum-based markers are relatively non-invasive, and can be conveniently used to monitor disease progress and supplement histopathological approaches. Therefore, several serum molecular markers for diagnostic or prognostic use in OSCC are currently under intense investigation, such as CCL2/CCL3, ß-carotene, CYFRA21-1 and growth differentiation factor 15 [16–19]. In addition to these soluble molecular markers, serum microRNA such as miRNA-31 has been proposed as a candidate biomarker for diagnostic or prognostic use in OSCC [20]. In this study, we suggested that serum Gas6 could become a new biomarker for diagnostic and prognostic use in OSCC patients.

Gas6 was identified due to its upregulation in fibroblasts under growth-arrest conditions [21, 22]. Gas6 could exert pleiotropic functions in biological processes. For example, Gas6 could promote platelet aggregation in the late phase of thrombus formation [23, 24]; Gas6 also could increase erythropoiesis and enhance leukocyte extravasation under inflammatory condition [25]. As a ligand, Gas6 could bind to the TAM receptor tyrosine kinases including Tyro-3, Axl and Mer, but the binding affinity of Gas6 is different (Axl>Tyro-3>Mer). It has been reported that Gas6 is overexpressed in different human cancers when compared with normal tissue [26–28]. Although no relevant research concerning the role of Gas6 in OSCC has been previously reported, several studies have shown that the Gas6/Axl signaling pathway plays a vital role in OSCC. Lee Ch, et al. found that the expression of Axl was gradually increased from normal epithelium to dysplasia to OSCC tissue, and Axl expression was also correlated with lymph node status and clinical stage. OSCC patients with high expression of Axl have a poorer prognosis than those with low Axl expression [29]. In concordance with these results, our data showed that elevated Gas6 level was related to lymph node metastases, TNM stage, prognosis, and so on. Taken together, these results suggest that the Gas6/Axl axis may be involved in the development of OSCC [29]. Gas6/Axl signaling activation could increase expression of mesenchymal markers when OSCC cells are co-cultured with tumor-associated macrophages. Under these conditions, inactivation of Gas6 may decrease the invasion/migration ability of OSCC cells [30] and Gas6 could increase proliferation and survival of cancer cells [12, 31]. Our study also found that Gas6 may promote the migration and invasion ability of OSCC cells.

For other types of squamous cell carcinoma, researchers have found Gas6/Axl signaling overexpression, for example, in human esophageal squamous cell carcinoma samples (ESCC) and ESCC cell lines. Inhibition of Axl gene expression may decrease cell survival, proliferation, migration, and invasion *in vitro* and tumor growth *in vivo*. Additionally, they have found that repression of Axl expression could lead to inhibition of NF-κB signaling, induce GSK3β activity and block ESCC cell proliferation in an Axl-dependent manner [32]. Whether Gas6 has a similar effect on NF-κB signaling or cell proliferation in OSCC cells needs further investigation. The Gas6/Axl pathway has been identified as promoting cancer cell invasion in several cancers and several mechanisms have been proposed. For example, Gas6/Axl signaling may activate the expression of metalloperoteinase-9 to facilitate tumor invasion through the coordination of ERK signaling, NF-kB and Brg-1 activation [33]. In cancer cells, EMT is a complex process, which may be regulated by multiple transcription factors such as Slug, Zeb1 and Zeb2 [34]. One previous study reported that transcription factor Slug was a target molecule of the Gas6/Axl pathway in HCC cells, suggesting Slug is essential for the induction of tumor invasion by Gas6 [35]. Although we found Gas6 could induce the EMT process and promote metastatic capacity of OSCC cells, we did not explore the accurate mechanism of Gas6-mediated metastases in OSCC cells. Investigations into the role of Gas6 in metastases in OSCC cells are eagerly awaited.

There were several limitations to our study. First, the number of OSCC patients was relatively small. When analyzing diagnostic efficiency, the small sample size may have limited the accuracy and reliability of the results. Second, the source of high serum Gas6 was not very clear. We found higher Gas6 expression in OSCC tumor tissue, but whether the higher serum Gas6 level is attributable to Gas6 overexpression in OSCC tumor tissue also needs further exploration. Third, we only focused on the effect of Gas6 on the EMT process and cell metastases *in vitro*, but this effect *in vivo* was not shown. Therefore, all these need further investigation.

In conclusion, we report that Gas6 level is significantly increased in OSCC patients, in serum and tumor tissue, and elevated serum Gas6 is related to late TNM stage and poorly differentiated tumors. A high Gas6 level may predict nodal metastases, late cancer stage and reflect poor prognosis in OSCC patients. Our study also confirmed that Gas6 is involved in the EMT process and promotes the metastatic capacity of OSCC cells. Taken together, these results suggest that Gas6 may be a candidate biomarker for diagnostic and prognostic use in OSCC patients.

## Supporting Information

S1 Table Characteristics of OSCC patients and normal controls. (DOCX)

S1 Fig Quantification of Gas6 expression between OSCC tumor tissue and tumor-adjacent tissue using densitometric analysis. (TIF)

S2 Fig Quantification of the individual proteins in YD38 cells using densitometric analysis. (TIF)

S1 File The Raw-Data of the present study. (XLSX)
