# Supplementary material for: Elevated Serum Gas6 Is a Novel Prognostic Biomarker in Patients with Oral Squamous Cell Carcinoma
Source: PLoS One. 2015 Jul 24;10(7):e0133940. doi: 10.1371/journal.pone.0133940 (PMC4514879; doi:10.1371/journal.pone.0133940)
Supplement: S1 Table — (DOCX) [file pone.0133940.s001.docx]

**Table S1:** Characteristics of OSCC patients and normal controls

| Characteristics | OSCC  case  (n=128) | Normal control  (n=145) | P value |
| --- | --- | --- | --- |
| Age (Year) | 53.4±10.4 | 52.6±9.7 | 0.516**^┎^** |
| Gender (n, %) |  |  |  |
| Male | 94 (73.4) | 103 (72.8) |  |
| Female | 34 (26.6) | 42 (27.2) | 0.380**^┝^** |
| Tobacco Smoking (n, %) |  |  |  |
| Non-Smoker | 37 (28.9) | 79 (53.4) |  |
| Smoker^┯^ | 91 (71.1) | 66 (46.6) | <0.01 |
| Alcohol Drinking (n, %) |  |  |  |
| Non-Drinker | 42 (32.8) | 93 (65.0) |  |
| Drinker^╁^ | 86 (67.2) | 52 (35.0) | <0.01 |
| TNM Stage |  |  |  |
| I/II | 53 (41.4) |  |  |
| III/IV | 75 (58.6) |  |  |
| Tumor Differentiation |  |  |  |
| Well | 42 (32.8) |  |  |
| Moderate | 48 (37.5) |  |  |
| Poor | 38 (29.7) |  |  |

**^┎^** :Statistical analysis performed with an un-paired t test;

**^┝^** : Statistical analysis performed using Pearson's Chi-squared test;

^┯:^ Smoker included: former smokers and current smokers;

^╁:^ Drinker included: former drinkers and current drinkers;
