# Supplementary figures and images for: Elevated Serum Gas6 Is a Novel Prognostic Biomarker in Patients with Oral Squamous Cell Carcinoma
Source: PLoS One. 2015 Jul 24;10(7):e0133940. doi: 10.1371/journal.pone.0133940 (PMC4514879; doi:10.1371/journal.pone.0133940)

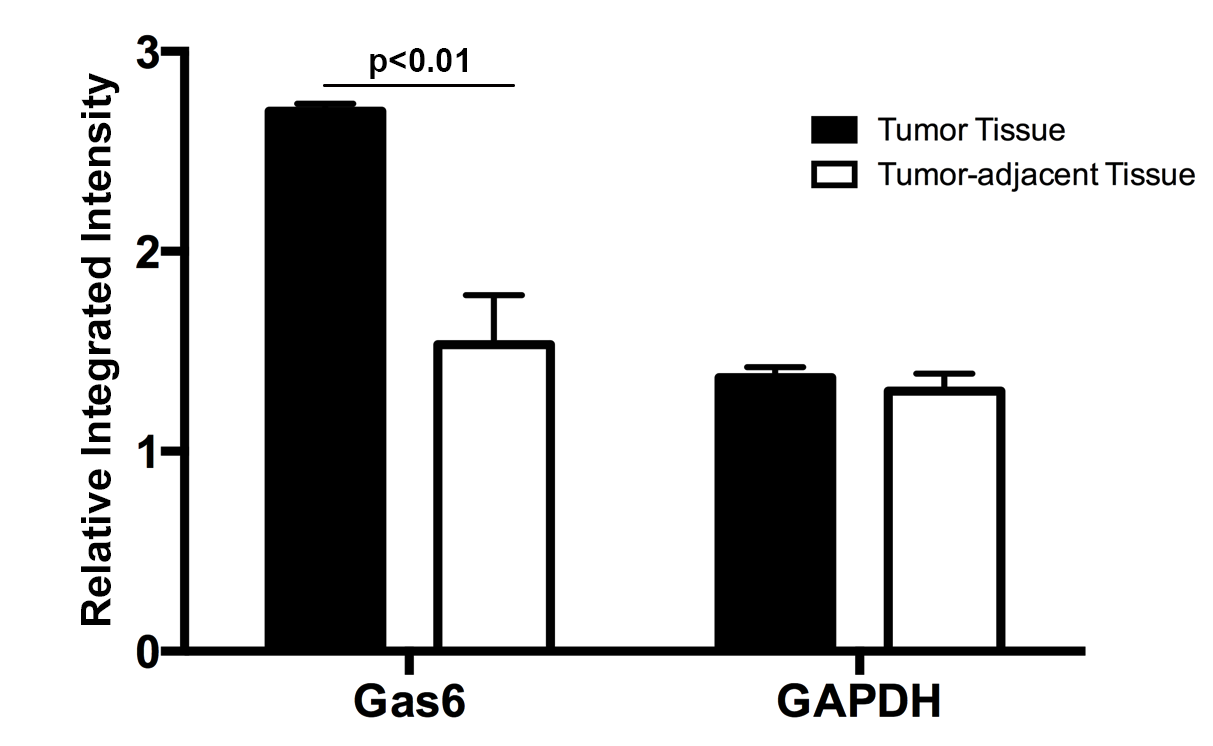

Supplement: S1 Fig — (TIF) [file pone.0133940.s002.tif]

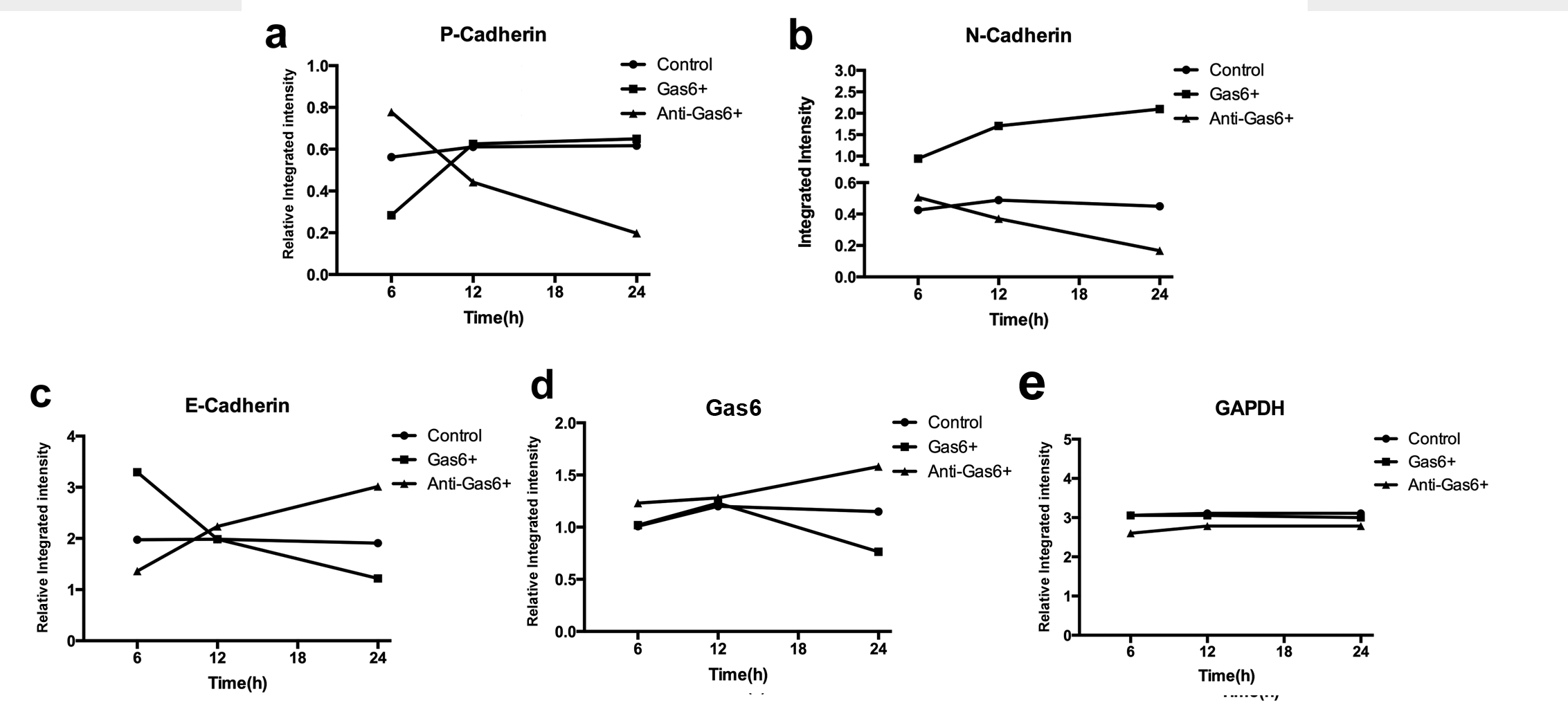

Supplement: S2 Fig — (TIF) [file pone.0133940.s003.tif]
